# Profile of the Lower Respiratory Tract Microbiome in Human Immunodeficiency Virus/Acquired Immunodeficiency Syndrome and Lung Disease

**DOI:** 10.3389/fmicb.2022.888996

**Published:** 2022-06-23

**Authors:** Zhen Chen, Ya Tian, Yu Wang, Hongxin Zhao, Chen Chen, Fujie Zhang

**Affiliations:** ^1^Savaid Medical School, University of Chinese Academy of Sciences, Beijing, China; ^2^Affiliated Beijing Ditan Hospital, Capital Medical University, Beijing, China; ^3^Affiliated Beijing Shijitan Hospital, Capital Medical University, Beijing, China

**Keywords:** HIV, CD4, next-generation sequencing, lower respiratory tract, bronchoalveolar lavage, microbiota

## Abstract

Once an human immunodeficiency virus (HIV)-infected individual enters the onset period, a variety of opportunistic infections may occur, affecting various systems and organs throughout the body, due to the considerable reduction in the body’s immune function. The objectives of this study were to explore the relationship between immune status and microbial communities in the lungs of individuals with HIV infection. A total of 88 patients with lung disease [80 (91%) HIV-positive and 8 (9%) HIV-negative] were enrolled in our study between January and July 2018, and 88 bronchoalveolar lavage fluid (BALF) samples were obtained during bronchoscopy. In this cross-sectional study, we investigated differences in the pulmonary microbiome of patients with HIV who had different immune statuses. The diversity of bacteria in the lungs of HIV-positive individuals was lower than that in HIV-negative individuals (*p* < 0.05). There was a significant difference in the composition and distribution of bacteria and fungi between the HIV-positive and HIV-negative groups (*p* < 0.01). The number of fungal species in the BALF of HIV-positive patients was higher than in HIV-negative patients. The diversity of bacteria and fungi in the BALF of HIV-positive patients increased with decreasing CD4 T-cell counts. Linear regression analysis showed that Pneumocystis (*R*^2^ = 6.4e−03, *p* < 0.05), Cryptosphaeria (*R*^2^ = 7.2e−01, *p* < 0.05), Candida (*R*^2^ = 3.9e−02, *p* < 0.05), and Trichosporon (*R*^2^ = 7.7e−01, *p* < 0.05) were negatively correlated with CD4 counts (*F*-test, *p* < 0.05). The samples collected from HIV-positive patients exhibited a different pattern relative to those from the HIV-negative group. Differences in host immune status cause differences in the diversity and structure of lower respiratory tract microorganisms.

## Introduction

In the past, based on culture-dependent techniques, it was thought that the lungs of healthy individuals were sterile. The primary tool for identifying pathogens responsible for lung infections was bacteriology-based culture (Muggeo et al., 2021). With advances in next-generation sequencing technology, researchers have found that, even in healthy individuals, the lungs are not as sterile as was once thought. Recent studies have shown that there is a unique microbiome in the lungs, which differs considerably between healthy individuals and those with respiratory diseases (Man et al., 2019; Naidoo et al., 2019). The lung flora is susceptible to environmental influences and has significant individual differences, but the correlation between the number of inflammatory cytokines and the lung flora has been confirmed in healthy mice, and the correlation between inflammatory cytokines and the lung flora is higher than that of intestinal bacteria (Dickson et al., 2018). Interactions between the host or through the immune system and the microbiome reveal potential mechanisms by which microbes affect respiratory health (Pattaroni et al., 2022). Studies have shown that the interaction between the environment and the host plays a certain role in the occurrence and development of idiopathic pulmonary fibrosis (IPF). In particular, microbial infection factors play an important role in the pathogenesis and progression of IPF. Respiratory dysbiosis is closely associated with IPF, but association does not equal causation (Segal and Molyneaux, 2019). In a prospective cohort study, factors such as the composition and function of the lower airway microbiota, and the host’s lower airway transcriptome characteristics were associated with clinical outcomes (Sulaiman et al., 2021).

These findings suggest that some lung diseases are related to changes in the lung microbiome. In individuals with chronic human immunodeficiency virus (HIV) infection who are not on treatment with antiretroviral agents, as their CD4+ T-lymphocyte count decreases they become vulnerable to a multitude of infections that rarely occur in an immunocompetent host, hence they are termed “opportunistic infections” (Tan et al., 2012). Among HIV-related pulmonary complications, opportunistic pneumonia is the leading cause of morbidity and mortality and a common reason for referral to a respiratory specialist for diagnostic evaluation and treatment (Shebl et al., 2010; Sigel et al., 2012, 2017). For those with access to antiretroviral therapy (ART), the spectrum of lung disease has shifted from acute opportunistic infections, which can lead to death, to chronic lung disease (Cribbs et al., 2020).

Pathogen identification in opportunistic infections is always difficult and is a critical issue faced by infectious disease clinicians. The low detection rate when using conventional culture methodology, especially for fastidious organisms, makes precision diagnosis challenging in most patients (Li et al., 2018). Culture-independent techniques, such as serologic assays and nucleic acid amplification tests, have proven useful for broadening the scope of detectable pathogens, but prior knowledge is necessary, which is sometimes impractical due to the complicated pathogen spectrum resulting from, for example, the popularity of international travel. Previous reports have suggested that up to 60% of cases are treated with no pathogen detected, despite the comprehensive testing methods available (Rhodes et al., 2010; Özçolpan et al., 2015; Schlaberg et al., 2017; Miao et al., 2018). Failure to obtain a specific and timely diagnosis may delay appropriate antimicrobial therapy, lead to unnecessary broad-spectrum antibiotic use and encourage antimicrobial resistance, and increase healthcare costs (Sartelli et al., 2017; Miao et al., 2018).

Amplicon sequencing is an unbiased method that, in theory, can detect all pathogens in a clinical sample; it is particularly suitable for complex infectious diseases, emerging infectious diseases, and atypical etiologies (Goldberg et al., 2015; Graf et al., 2016; Schlaberg et al., 2017; Zhang et al., 2020; Gu et al., 2021; Wang et al., 2021). As this technology evolves, amplicon sequencing may have the potential to become a routine diagnostic test, partly replacing traditional detection techniques, due to its advantages in sensitivity, speed, and cost.

However, the question of whether there is a relationship between immune status and the pulmonary microbiome has not yet been addressed. In this study, we used amplicon sequencing to explore this question. This study systematically investigated the composition and changes of the respiratory microbiome in HIV-infected individuals with different immune status in a large cohort sample. We found differences in the microbial community structure in the lungs of HIV-infected and non-infected individuals. We found that Pneumocystis, Candida and some other fungal species were significantly increased in a state of immunosuppression. At the same time, with the gradual loss of immune function, the types of microorganisms in the lungs of the human body increase. These findings suggest that there is a certain relationship between the community structure of lung microbes and immune status.

## Materials and Methods

### Patient Recruitment and Sample Collection

We recruited 88 patients infected with HIV, who were admitted to Ditan Hospital, Beijing, China, between January and July 2018, according to strict inclusion and exclusion criteria, and collected clinical data for follow-up analysis. As participants in this study, the patients underwent bronchoscopy, and 10 ml of bronchoalveolar lavage fluid (BALF) was set aside for microbiome analysis. Bronchoalveolar lavage is performed on the most involved lung segments on the chest radiograph. The median time to bronchoscopy following admission was 1 day [interquartile range (IQR), 1–3 days]. This study received approval from the Medical Ethics Committee of our hospital, and all patients signed informed consent forms before undergoing bronchoscopy. All participants provided written informed consent to participate in the survey and biomarker testing. At the same time, we used dd water as negative control, and the taxa results are in Table 1.

**TABLE 1:** Clinical features of participants.

	G1 (*n* = 45)	G2 (*n* = 26)	G3 (*n* = 9)	Non HIV (*n* = 8)	*p* - value
Age	33.00 (29.00–45.00)	42.50 (31.00–51.25)	39.00 (37.00–49.00)	66.50 (53.50–71.00)	<0.001
Gender = male	43 (95.6%)	22 (84.6%)	7 (77.8%)	6 (75.0%)	0.166
**Smoking**					0.2
giveup	3 (7.9%)	2 (8.3%)	2 (33.3%)	2 (33.3%)	
no	26 (68.4%)	19 (79.2%)	3 (50.0%)	2 (33.3%)	
yes	9 (23.7%)	3 (12.5%)	1 (16.7%)	2 (33.3%)	
Alcohol	7 (18.4%)	3 (12.5%)	0 (0.0%)	4 (66.7%)	0.012
CD3+/CD45+–%	6.9e+01 (11.50)	78.08 (9.51)	75.11 (12.21)	68.54 (8.92)	0.012
CD3+–cells/uL	4.4e+02 (310.25–716.75)	684.50 (466.75–1,241.75)	1,215.00 (1,061.00–1,261.00)	997.00 (896.00–1,669.50)	<0.001
CD8+/CD45+–%	6.4e+01 (10.40)	64.22 (16.22)	49.93 (13.89)	31.32 (6.55)	<0.001
CD8+–cells/uL	4.2e+02 (287.00–653.75)	576.50 (391.00–1,097.00)	754.00 (598.00–996.00)	364.00 (358.00–855.50)	0.021
CD4+/CD45+ - %	1.6e+00 (0.89–3.23)	8.65 (6.62–12.53)	25.00 (16.55–29.08)	33.51 (31.42–40.46)	<0.001
CD4+ - cells/uL	1.0e+01 (6.00–22.00)	86.00 (64.50–127.25)	364.00 (268.00–440.00)	606.00 (524.00–812.00)	<0.001
CD45+ - cells/uL	6.9e+02 (475.75–894.50)	923.00 (622.25–1,508.75)	1,728.00 (1,186.00–2,092.00)	1,320.00 (1,299.50–2,396.00)	<0.001
CD4+/CD8+ - %	2.0e–02 (0.01–0.05)	0.15 (0.09–0.21)	0.57 (0.22–0.70)	1.01 (0.88–1.14)	<0.001
VL - copies/mL	1.7e+05 (113,417.00–4e+05)	430.00 (40.00–73,112.00)	20.00 (0.00–17,759.25)	–	<0.001
**ART**					
3TC+TDF+EFV	37 (92.5%)	16 (66.7%)	3 (50.0%)	–	
3TC+TDF+KLC	1 (2.5%)	2 (8.3%)	1 (16.7%)	–	
AZT+3TC+EFV	0	1 (4.2%)	1 (16.7%)	–	
AZT+NVP+3TC	0	1 (4.2%)	0	–	
DTG+FTC+TDF	0	1 (4.2%)	0	–	
KLZ+3TC+DTG	0	1 (4.2%)	0	–	
TDF+3TC+LPV/r	2 (5.0%)	2 (8.3%)	1 (16.7%)	–	
WBC - 109/L	4.6e+00 (2.91–6.51)	4.89 (3.67–6.53)	5.42 (3.80–6.86)	5.44 (5.09–9.99)	0.326
PCT - ng/mL	7.0e–02 (0.05–0.13)	0.05 (0.05–0.07)	0.08 (0.06–0.32)	0.05 (0.05–0.05)	0.36
CRP - mg/L	1.4e+01 (4.20–42.90)	11.40 (5.65–30.70)	6.40 (1.80–41.20)	12.40 (4.00–28.20)	0.909

*VL, viral load; ART, antiretroviral therapy; 3TC, (−)-2′,3′-dideoxy-3′-thiacytidine; TDF, tenofovir disoproxil fumarate; KLC, keiva lei cadena; AZT, azidothymidine; EFV, efavirenz; NVP, nevirapine; DTG, dolutegravir; FTC, emtricitabine; LPV/r, lopinavir/ritonavir; WBC, white blood count; PCT, procalcitonin; CRP, C-reactive protein.*

### DNA Extraction, Library Construction, and Sequencing

DNA extraction experiments and amplicon sequencing were performed at the Institute of Infectious Diseases, Beijing Ditan Hospital, affiliated to Capital Medical University. We used full-length 16S rDNA and internal transcribed spacer (ITS) rDNA for sequencing, respectively. DNA was extracted using a MagNA Pure LC 2.0 system and a MagNA Pure LC Total NA Isolation kit (Roche, Mannheim, Baden-Württemberg Land, Germany), in accordance with the manufacturer’s instructions, and quantified using a Quant-iT PicoGreen dsDNA assay kit (Invitrogen, Eugene, Oregon, United States). Polymerase chain reaction (PCR) amplification of the full length region was performed with the following primers containing Illumina adapter sequences and dual-index barcodes to tag each sample: 341F 50-CCTACGGGNGGCWGCAG-30 and 805R 50-GACTACHVGGGTATCTAATCC-30. The PCR reaction conditions were as follows: 95°C for 3 min, followed by 25 cycles of denaturation at 95°C for 30 s, annealing at 65°C for 30 s, extension at 72°C for 30 s, and a final extension step at 72°C for 5 min. PCR products were then cleaned using AMPure XP beads (item no. A63882; Beckman Coulter Inc., Fullerton, CA, United States). The amplicon sequencing libraries were constructed in accordance with the 16S Metagenomic Sequencing Library Preparation (Illumina, Inc., San Diego, CA, United States). Paired-end sequencing with a read length of 250 bp × 2 was performed on a MiSeq instrument (Illumina, Inc.) using a MiSeq v2 reagent kit (Illumina, Inc.).

### Raw Data Processing and Microbial Taxonomy Assignment

#### 16S OTU Table Generation

Quality control of raw sequencing data was performed using Fastqc (v0.11.9) (FastQC, 2015). For bioinformatic processing of the MiSeq results, raw FASTQ files were de-multiplexed and quality filtered using QIIME (Quantitative Insights Into Microbial Ecology, v1.9.1) (Caporaso et al., 2010). The sequencing data were then quality filtered using USEARCH’s fastq filter (v7.0.1001) to remove reads with more than two expected errors. The strategy of open-reference out-picking was used for cluster analysis of the sequencing data. This strategy automates species annotation of OTUs by referring to species taxonomic information in the database. At the same time, the de novo OTU picking method was used to cluster those sequences without corresponding reference sequences in the database. The Greengenes Database (May 2013) was used to assign classifications to OTUs. The database includes a total of 1,262,986 16S ribosomal RNA (rRNA) sequences. The results were manually organized and classified using the commonly used seven-class classification method for easy understanding and reading. QIIME was used to construct the phylogenetic trees. OTUs were filtered by: (1) removing OTUs with contaminants commonly found in BAL samples using the negative control sample as a reference, (2) removing any OTUs in BAL samples with fewer than 10 reads, (3) OTUs with reads less than 1/5000 of the total reads of all samples were removed (Shenoy et al., 2019).

#### Internal Transcribed Spacer OTU Table Generation

Internal transcribed spacer OTUs were generated using a similar strategy to that outlined above, with the following modifications and annotations: (1) chimeras were removed and taxa assigned using DADA2 (v1.14) *via* the UNITE database (Callahan et al., 2016, Nilsson et al., 2019); (2) no phylogenetic tree was generated; (3) no NTC samples; (4) OTUs with reads less than 1/1000 of the total reads of all samples were removed.

### Statistical Analysis

Diversity indices were calculated using QIIME and DADA2. The Bray–Curtis distance was used to find the principal coordinates, and PERMANOVA was used to check the accuracy of the principal coordinates analysis (PCoA) results. The above calculations were performed using the vegan package (Dixon, 2003) in the R language (v.4.0.2). Relative log expression (RLE): similar to TMM, this normalization method is based on the hypothesis that the most genes are not DE. For a given sample, the RLE scaling factor is calculated as the median of the ratio, for each gene, of its read counts over its geometric mean across all samples. By assuming most genes are not DE, the median of the ratio for a given sample is used as a correction factor to all read counts to fulfill this hypothesis. This normalization method is included in the DESeq and DESeq2 Bioconductor packages (Anders and Huber, 2010; Love et al., 2014).

## Results

Between 16 January and 5 July 2018, 80 patients with HIV infection who were admitted to Ditan Hospital were included in our study cohort, based on their CD4+ cell counts. For comparison, we also recruited eight HIV-negative patients diagnosed with pulmonary infectious diseases based on clinical experience by experienced clinicians. The patients’ clinical characteristics are shown in Table 1. The median CD4 count of the HIV-positive group was 39 cells/μl (IQR 9–94), the median plasma HIV RNA concentration was 5.1 log10 copies/ml (2.5–5.4), and 11/74 (15%) patients had HIV RNA concentrations higher than 500,000 copies/ml. The HIV-positive patients were divided into three groups according to WHO guidelines: group I (G1, CD4 < 50 cells/μl), group II (G2, 50 < CD4 < 200 cells/μl), and group III (G3, CD4 > 200 cells/μl). As the CD4 cell count decreased, CD3, CD8, and CD45 decrease in HIV-positive patients, while the viral load (VL) increased (Wilcoxon, *p* < 0.001, Table 1).

### Alterations in the Profile of Microbiota in the Lungs of Human Immunodeficiency Virus-Infected Patients

The current paradigm for diagnosing infections relies on the physician formulating a differential diagnosis on the basis of a patient’s history, clinical presentation, and imaging findings, followed by serial laboratory testing. The most commonly used method for profiling microbial communities is sequencing of the 16S rRNA gene and ITS regions for bacteria and fungi, respectively (Bukin et al., 2019; Johnson et al., 2019). The universal distribution and conserved nature of the 16S rRNA and ITS genes means they are well-established genetic markers used for bacterial and fungal identification and classification.

**FIGURE 1:**
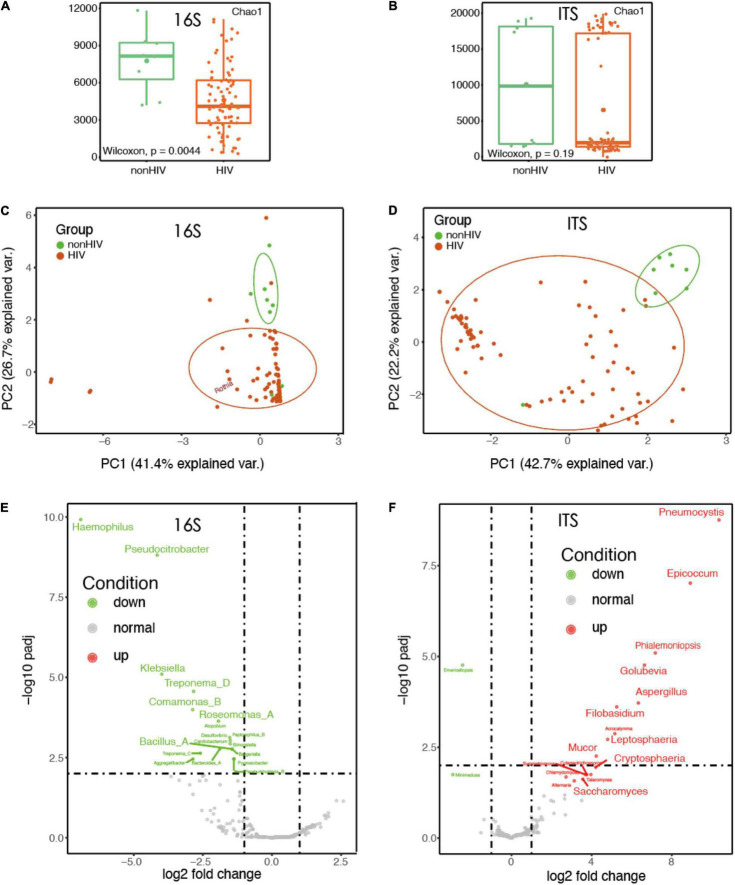
The diversity of HIV-infected microbiota decreased and the structure of the bacteria changed. **(A)** Comparison of bacterial alpha diversity in BALF at the genus level between HIV-negative individuals and HIV-positive individuals treated with HAART. **(B)** Comparison of fungal alpha diversity in BALF fluid at the genus level between HIV-negative individuals and HIV-positive individuals treated with HAART. **(C)** Principal coordinates analysis (PCoA) using a Bray–Curtis dissimilarity of BALF sample (16S rRNA; *n* = 88) bacterial community profiles, representatively rarefied to 38,721 reads/sample. **(D)** Principal coordinates analysis (PCoA) using a Bray–Curtis dissimilarity of BALF sample (ITS rRNA; *n* = 88) fungal community profiles, representatively rarefied to 58,677 reads/sample. **(E)** Volcano plots of genus-level taxonomic bins in the 16S rRNA gene datasets. The *x*-axes display the fold changes in relative abundance (log 2). **(F)** Volcano plots of genus-level taxonomic bins in the ITS rRNA gene datasets. The *x*-axes display the fold changes in relative abundance (log 2).

The data of 25 samples were randomly selected to draw a dilution curve, and the results showed that the sequencing depth met the analysis requirements (Supplementary Figure 1). The top five phylum-level species in the 16S data were Firmicutes, Proteobacteria, Actinobacteriota, Bacteroidota, and Fusobacteriota. The top five phylum-level species in the ITS data are Chytridiomycota, Glomeromycota, Mortierellomycota, Basidiomycota, and Ascomycota. Bacterial microbiota profiles were generated using whole 16S rRNA amplicon sequencing for lower airway samples, while BAL fungal microbiota composition was investigated using whole ITS rRNA sequencing. By 16S rDNA sequencing technology, we detected 1291 OTUs, of which 983 were identified as bacterial species. At the same time, 670 OTUs of fungi were found, and 432 fungus were identified. Alpha diversity based on filtered and normalized reads indicated that HIV-positive patients had less bacterial species diversity at the genus level in their alveolar lavage fluid, based on the Chao1 index (Wilcoxon, *p* < 0.05). However, a similar pattern was not observed in the analysis of fungal alpha diversity (Wilcoxon, *p* > 0.05; Figure 1A,B). Comparison of bacterial beta-diversity in BAL at the OTU level between uninfected and HIV-infected individuals treated with ART using principal coordinate analysis (Bray–Curtis) revealed that the HIV-positive population remained significantly different compared to BAL from uninfected individuals even on therapy (Figure 1C, PERMANOVA, *p* < 0.05). To describe the fungal variation in the composition of samples between groups, PCoA was also used with the fungal datasets. Similar to the results for bacteria, the fungal colony structure of HIV-positive individuals differed greatly from that of HIV-negative individuals. However, different from the results of bacterial PCoA, even within HIV-infected individuals, the differences were still significant (Figure 1D, PERMANOVA, *p* < 0.05).

We observed a very interesting phenomenon, which was that in the 16S rRNA dataset of BALF sample of HIV-positive patients, some common pathogenic bacteria, such as Klebsiella, Bacillus, and Haemophilus, were reduced to varying degrees (Figure 1E). Instead, a large number of fungi, such as Pneumocystis, Aureobasidium, Cystobasidium, and Saccharomyces, were increased in the samples of HIV-positive patients’ BALF; these fungi have mostly been reported to be likely to cause infection (Figure 1F).

### The Types of Microorganisms in the BAL of Human Immunodeficiency Virus-Positive Individuals Increase With Decreasing CD4 Count

**FIGURE 2:**
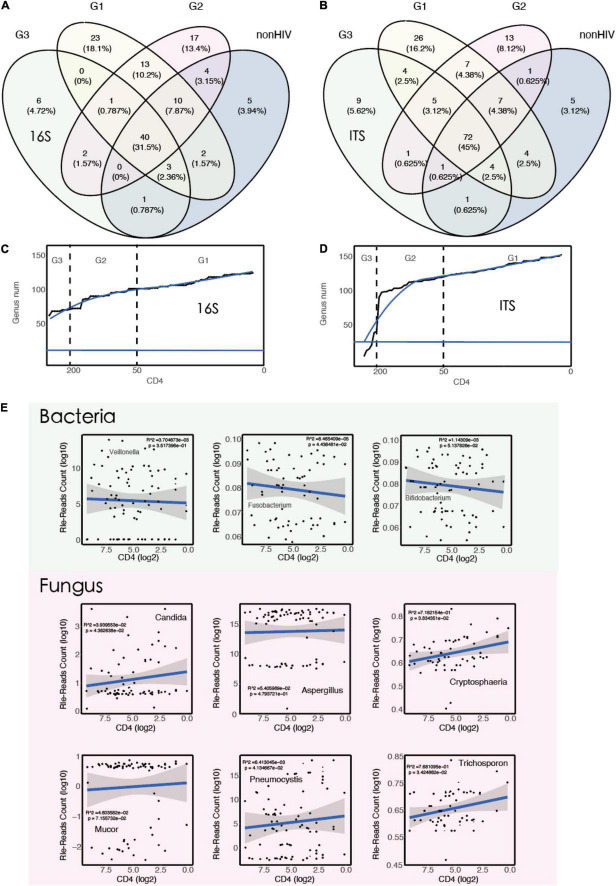
The types of microorganisms in the BALF of HIV-positive individuals increase with decreasing CD4 counts. **(A)** A Venn diagram showing bacterial genus distribution of 16S rRNA for each group (G1 vs. G2 vs. G3 vs. non-HIV). **(B)** A Venn diagram showing fungal genus distribution of ITS rRNA for each group (G1 vs. G2 vs. G3 vs. non-HIV). **(C)** Smooth line diagram of the change in the bacterial count target. **(D)** Smooth line diagram of the change in the fungal count target. **(E)** Linear model used to describe the relationship between species numbers and CD4 counts.

The RLE method was used to standardize the data. For bacteria, 16S rRNA identified a positive microorganism (to genus level) whose coverage rate scored fivefold greater than that of any others. For fungi, ITS identified a microorganism (to genus level) whose coverage rate scored twofold higher than that of any other fungus, because of its low biomass in DNA extraction (Miao et al., 2018). A Venn diagram was plotted and showed that in HIV-positive patients, the types of microorganisms in the BALF increased, including both bacteria and fungi. Moreover, as the immune function of HIV-positive patients continued to decline, more and more microorganisms appeared in the patients’ BALF. In addition, it is interesting to note that when CD4 cell counts dropped below 50 cells/μl, the BALF of HIV-positive patients showed a previously unseen pattern of species. There were 23 (18.1%) and 26 (16.2%) specific species of bacteria and fungi in the G1 group, respectively (Figures 2A,B).

The patients were ranked by CD4 count decline, and the number of newly emerged species compared with the previous patient was counted. The results showed that the number of species showed an upward trend as CD4 cell counts decreased. This finding also supports the aforementioned results (Figures 2C,D). To verify this result from the opposite direction, we randomly calculated 10,000 times, then simulated and counted the changes in the number of species, and the results did not show the increasing trend described above. In other words, the increasing number of microorganisms seen with the decrease in CD4 cell counts is not a common phenomenon. It only occurs when the immune function is suppressed, as in acquired immunodeficiency syndrome (AIDS) patients, as more pathogenic microorganisms appear in various parts of the body. The relationship between the immune system and the microbiota has been reported previously (Weng and Walker, 2013; Mirpuri et al., 2014).

To explore the association between microbiota abundance and immune status, we constructed a linear regression model using CD4 counts and standardized read counts and screened a subset of taxa based on previous studies (Wakefield et al., 1990; Abrahamsson, 2016; Routy et al., 2018; Lam et al., 2022). The results showed that with the decrease in CD4 counts, Fusobacterium (*R*^2^ = 8.5e−05, *p* < 0.05) decreased. At the same time, the abundance of fungi increased with the decrease in CD4 counts, especially some common fungi associated with lung infections, for example, Candida, Cryptosphaeria, Pneumocystis, and Trichosporon (Figure 2E). Pneumocystis carinii is a common pathogenic fungus that casuse in lung infections and has been frequently reported in HIV-positive patients (Balaan, 1990; Thomas and Limper, 2004).

**FIGURE 3:**
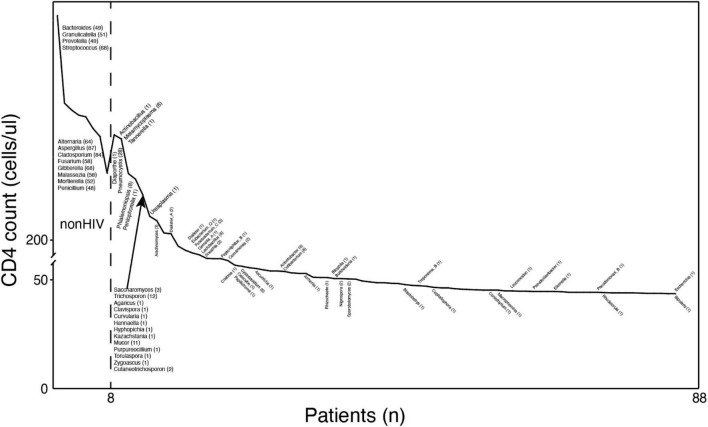
The types of microorganisms in the BALF of HIV-positive individuals increase with decreasing CD4 counts. Profiles of pathogenic microorganisms in HIV/AIDS patients under different immune states. The *y*-axis coordinate is the CD4 cell count. The *x*-axis coordinate is a patient at this CD4 value. The text near the curve indicates newly discovered microorganisms at this CD4 value. The numbers in parentheses are the numbers of samples containing the species.

Positive species judged positive by coverage rate were screened according to the aforementioned method and marked at their first occurrence in Figure 3. Species that infect humans and have been reported previously were retained in the graph (Bogaert et al., 2004; Kovacs and Masur, 2009; Hampton, 2011; Boussat et al., 2022).

Overall, these results suggest that changes in the flora of HIV-positive patients are associated with changes in immune status and that the loss of normal symbiotic flora may make patients more susceptible to secondary infection by other pathogens.

## Discussion

Infection with the human immunodeficiency virus type 1 (HIV-1) results in the progressive loss of immune function, marked by the depletion of CD4+ T-lymphocytes, leading to opportunistic infections and malignancies characteristic of AIDS (Kakuru et al., 2016). Although both host and viral determinants influence the rate of disease progression, the median time from initial HIV infection to the development of AIDS among untreated patients ranges from 8 to 10 years (Hansen et al., 2016). Clinical staging of HIV disease and the relative risk of developing opportunistic infections have historically relied on CD4+ T-lymphocyte counts. Although more recent studies have shown the importance of VL quantitation in determining the rate of disease progression, it is still useful to categorize HIV disease stages based on the degree of immunodeficiency (Eisinger et al., 2019). The host biological factors that determine disease severity and outcome in HIV-positive patients with pneumonia are not fully understood, and previous studies have focused more on the role of gut microbial communities (Ortiz et al., 2018; Wang et al., 2020). Reports suggest that the pulmonary microflora of HIV-positive patients with pneumonia is mainly composed of Prevotellaceae, Streptococcus, and Pseudomonadaceae (Twigg et al., 2017). Our study confirms previous reports that individual bacterial diversity decreases in HIV-positive patients with pneumonia but increases in population bacterial diversity. As we suspected, the lower respiratory tract microbiota was associated with HIV disease severity (CD4 cell count). This is an important finding, as there is increasing evidence that microorganisms play a role in regulating both local and distal mucosal immunity, as well as in responding to inflammatory responses to microbial infections.

A limitation of this study was that all patients in the cohort were given antibiotics, which may have interfered with their microbial community. Despite this limitation, this study is the first to reveal systemic perturbations of the microbiota and the relationship between respiratory microbiota composition and CD4 status in HIV-positive patients with pneumonia. This finding suggests that paying attention to respiratory flora and the development of new treatment strategies may improve the survival rate of HIV-positive patients with respiratory infection.

## Conclusion

This study systematically examined the respiratory tract mycobiome in a group of patients with lung disease. A strong relationship was observed between an individual’s lung microbiome and immune status. As CD4 counts decreased, the variety of bacteria in the lungs increased, but the quantity decreased. At the same time, the number and variety of fungi increased as an individual’s immune status worsened.

## Data Availability Statement

The datasets presented in this study can be found in online repositories. The names of the repository/repositories and accession number(s) can be found in the article/Supplementary Material.

## Ethics Statement

The studies involving human participants were reviewed and approved by the Ditan Hospital. The patients/participants provided their written informed consent to participate in this study.

## Author Contributions

FZ and CC conceptualized the survey. YT managed the data collection. ZC conducted the data cleaning, analysis, and manuscript writing. YW and HZ provided the additional analytic support. All authors critically reviewed and approved the final version of the manuscript.

## Conflict of Interest

The authors declare that the research was conducted in the absence of any commercial or financial relationships that could be construed as a potential conflict of interest.

## Publisher’s Note

All claims expressed in this article are solely those of the authors and do not necessarily represent those of their affiliated organizations, or those of the publisher, the editors and the reviewers. Any product that may be evaluated in this article, or claim that may be made by its manufacturer, is not guaranteed or endorsed by the publisher.

## Supplementary Material

Supplementary Figure 1 Sparse curve describing sequencing depth. The *x*-axis is richness, the *y*-axis is the number of sequence.

Supplementary Figure 2 Stacked plot depicting species distribution at the phylum level. The *x*-axis is the grouping information of the samples. The *y*-axis represents the relative abundance of species.

Supplementary Figure 3 Comparison of alpha diversity in BALF at the genus level between HIV-negative individuals and HIV-positive individuals treated with HAART.
