# Supplementary figures and images for: Profile of the Lower Respiratory Tract Microbiome in Human Immunodeficiency Virus/Acquired Immunodeficiency Syndrome and Lung Disease
Source: Front Microbiol. 2022 Jun 23;13:888996. doi: 10.3389/fmicb.2022.888996 (PMC9260662; doi:10.3389/fmicb.2022.888996)

A

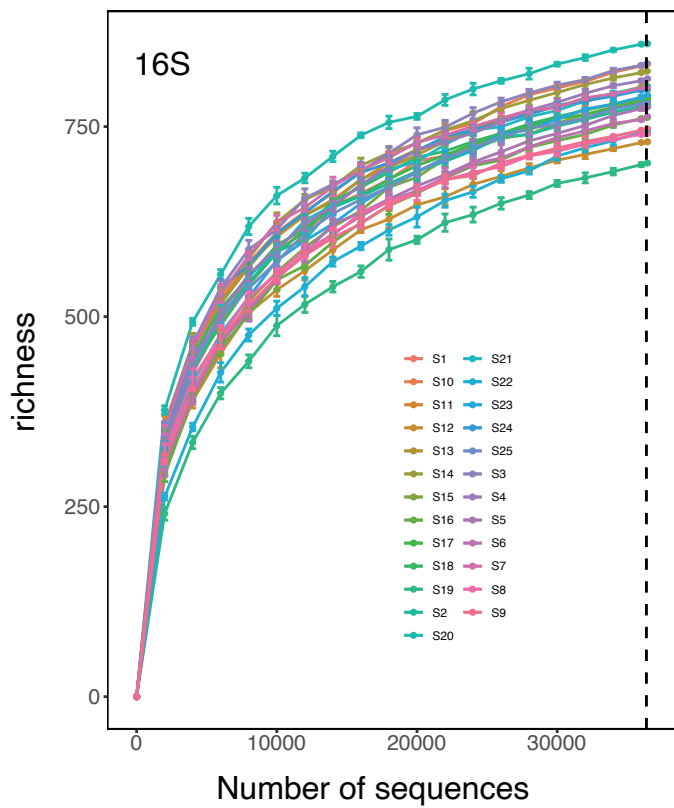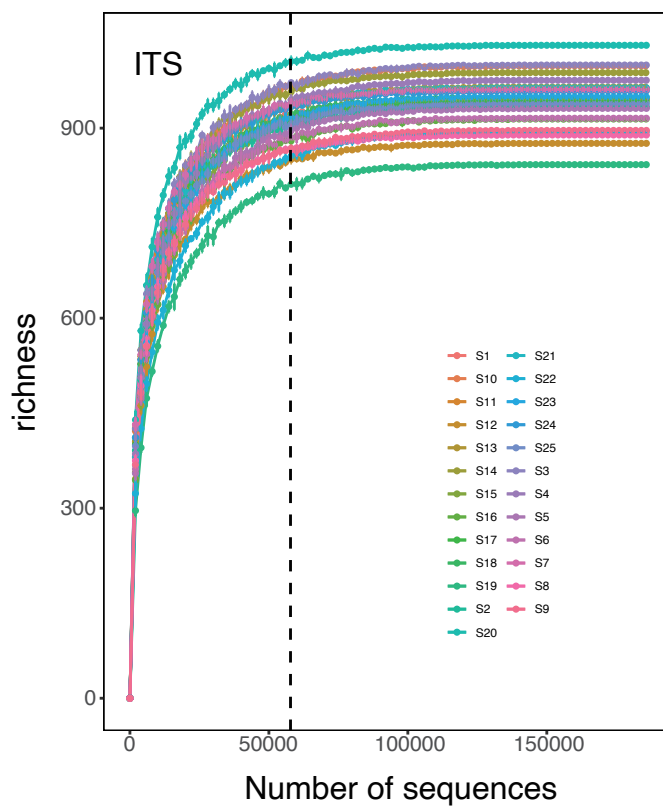

Supplement: Supplementary Figure 1 — Sparse curve describing sequencing depth. The x-axis is richness, the y-axis is the number of sequence. [file Image_1.pdf]

16S

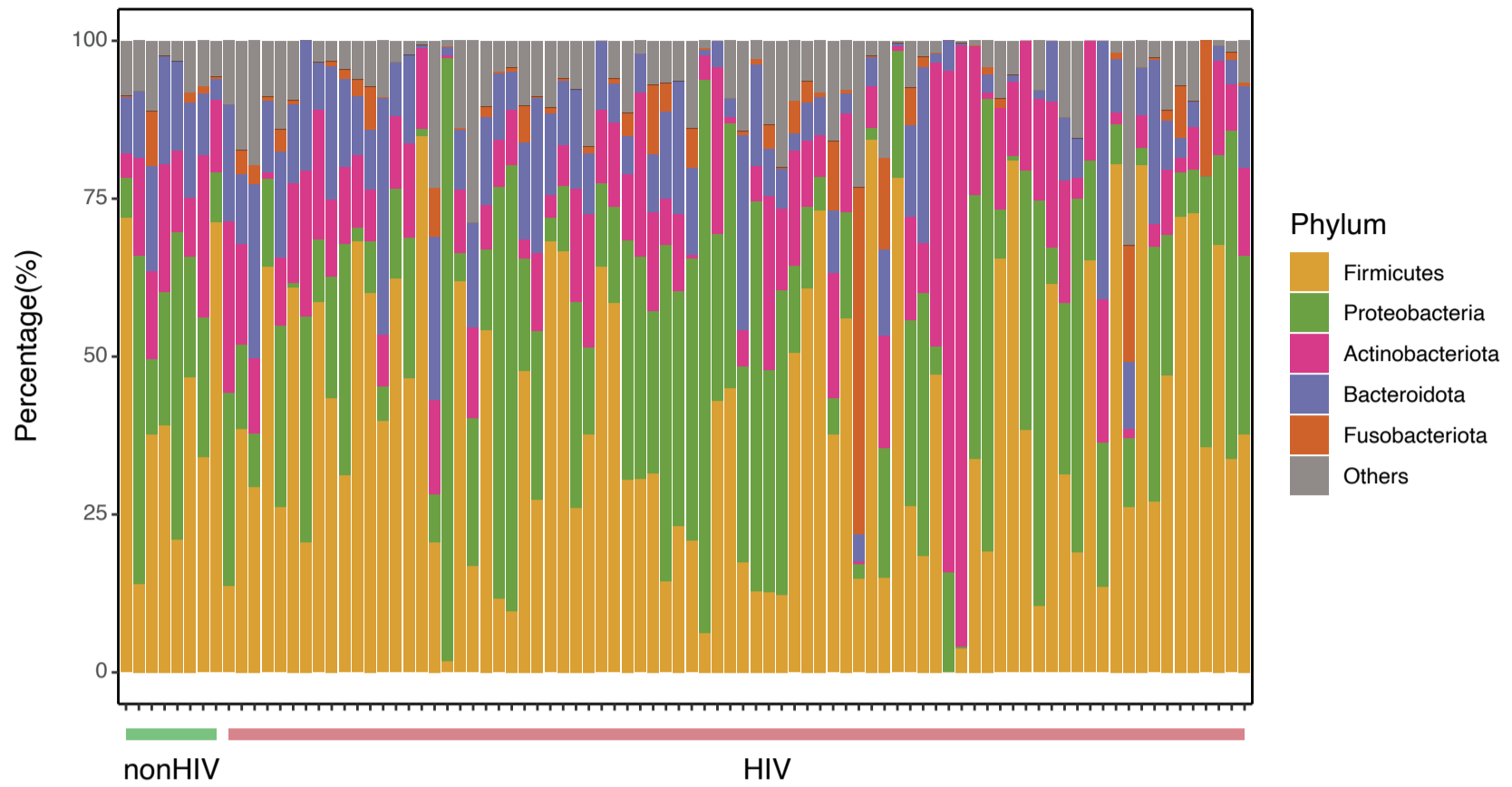

ITS

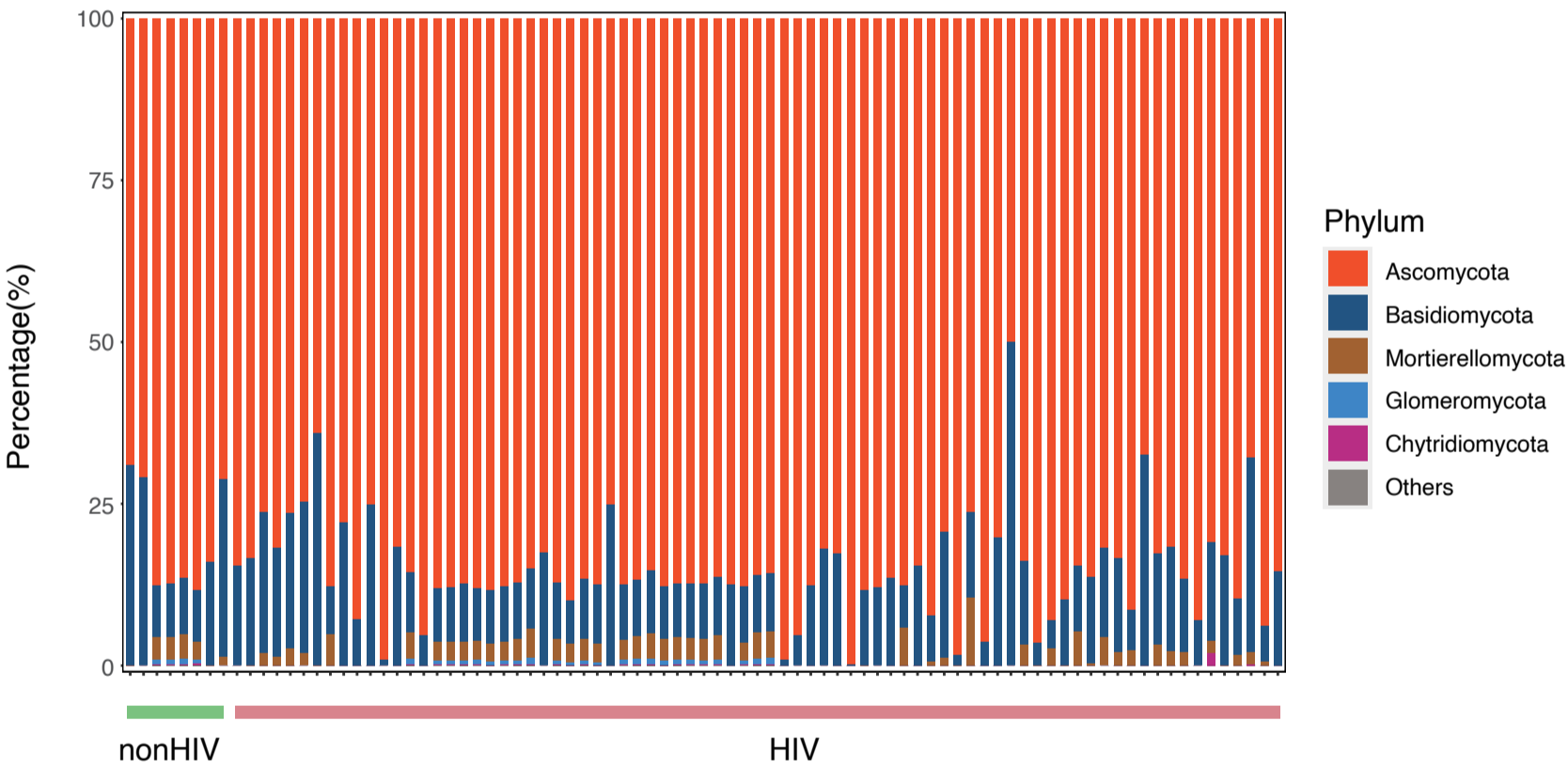

Supplement: Supplementary Figure 2 — Stacked plot depicting species distribution at the phylum level. The x-axis is the grouping information of the samples. The y-axis represents the relative abundance of species. [file Image_2.pdf]

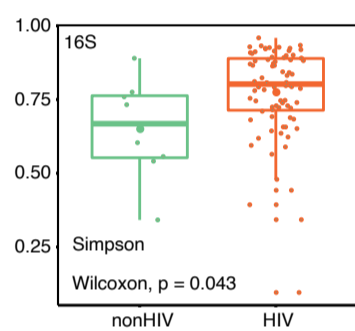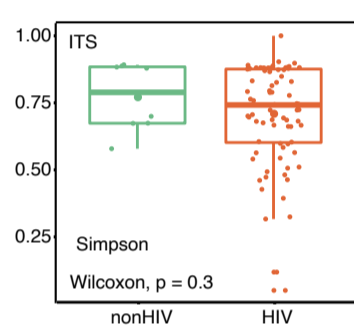

Supplement: Supplementary Figure 3 — Comparison of alpha diversity in BALF at the genus level between HIV-negative individuals and HIV-positive individuals treated with HAART. [file Image_3.pdf]
